# Nodular Basal Cell Carcinoma in an Unusual Groin Location: A Rare Presentation

**DOI:** 10.7759/cureus.54552

**Published:** 2024-02-20

**Authors:** Noura Seghrouchni, Nassira Karich, Asmae Aissaoui, Youssef Bouyahyaoui, Amal Bennani

**Affiliations:** 1 Department of Pathology, Faculty of Medicine and Pharmacy of Oujda, Mohammed First University of Oujda, Oujda, MAR; 2 Department of Dermatology, Faculty of Medicine and Pharmacy of Oujda, Mohammed First University of Oujda, Oujda, MAR

**Keywords:** case report, rare location, perineal region, groin region, basal cell carcinoma

## Abstract

Basal cell carcinoma is the most frequent skin malignancy with a constant rise in its incidence. It affects typically the head and neck of elderly patients. However, the literature in English shows its occurrence in many uncommon locations. In our work, we report a case of basal cell carcinoma occurring in the groin region in a 66-year-old male patient, with no particular medical history. We also discuss through a literature review, the characteristics of this common neoplasm when it occurs in the groin and in other atypical locations.

## Introduction

Basal cell carcinoma (BCC) is a prevalent skin cancer affecting millions in the US annually. While typically slow-growing, some lesions can be locally invasive and destructive. Although rare, metastasis to lymph nodes can occur, albeit with a low rate of 0.1% [1].

BCC typically develops on sun-exposed skin in elderly individuals, with the head and neck being the two most common locations, although it could occur anywhere. However, it is rarely presented on non-exposed skin [1].

Histologically, it could be classified into different subtypes: nodular, pigmented, superficial, infiltrative, sclerosing, fibroepithelioma of Pinkus, and polypoid [2].

## Case presentation

We report the case of a 66-year-old man, presenting with a slowly growing skin lesion of the right groin. No significant clinical personal or familial history was found.

**Figure 1:**
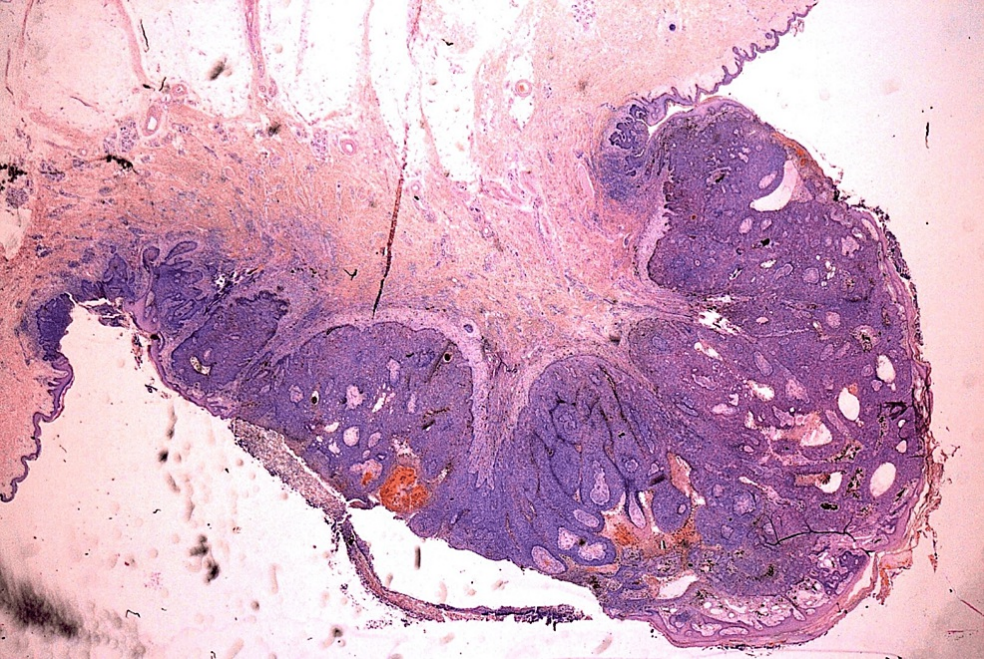
Low-magnification microphotograph displaying a nodular BCC comprised of well-defined, voluminous lobules composed of basaloid cells with a palisading arrangement at the periphery. BCC: Basal cell carcinoma.

Clinical examination revealed a 3x1.2x1.5 cm, flesh-colored nodular lesion. The surface was smooth with no ulceration (Figure 1).

**Figure 2:**
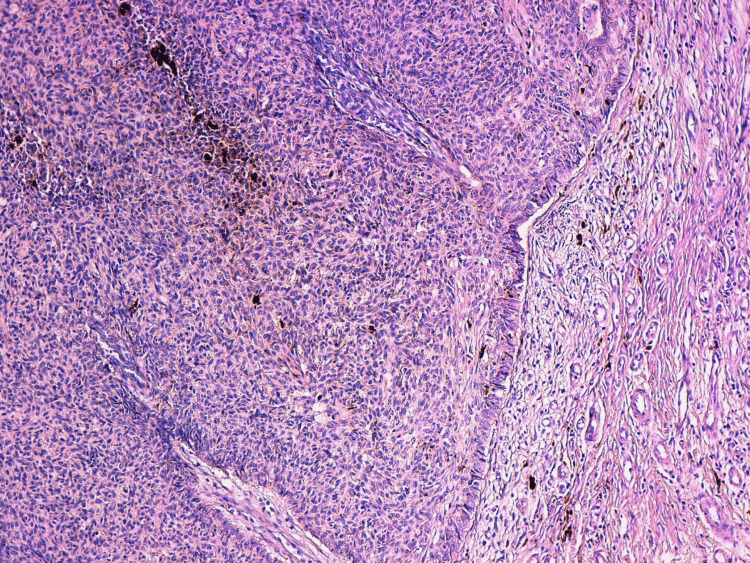
The proliferation is made of basaloid cells, with peripheral palisading and focal retraction artifacts.

**Figure 3:**
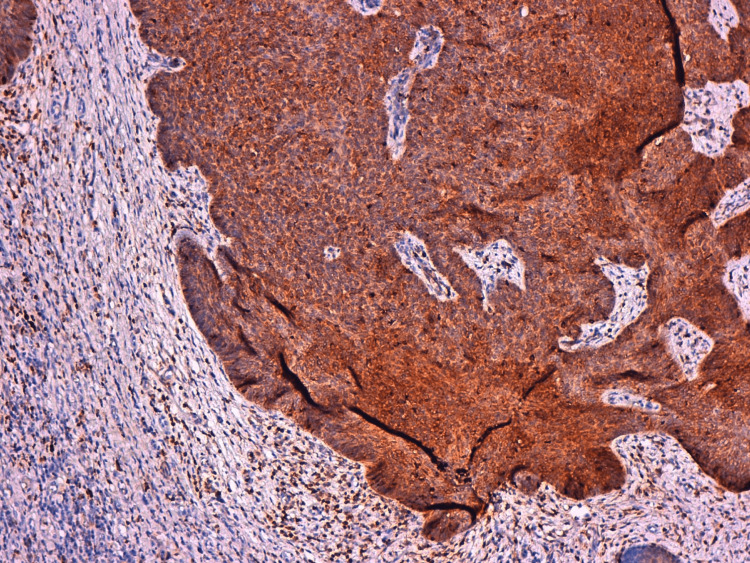
The neoplastic cells showed expression of BCL-2. BCL-2: B-cell lymphoma 2.

A resection of the lesion was performed. On the pathological level, we could observe a proliferation of basaloid cells with a high nucleocytoplasmic ratio extending from the epidermis to the dermis. These cells were organized in large nests and made a prominent peripheral palisading with many retraction artifacts that were focally filled with a myxoid material. Tumor cells also focally contain melanin pigments in their cytoplasm (Figures 2, 3).

On immunohistochemistry, tumor cells showed expression of B-cell lymphoma 2 (BCL-2) but no expression of epithelial membrane antigen (EMA).

The surgical margins were free of tumors.

The diagnosis of BCC was made, based on histological and immunohistochemical findings.

## Discussion

BCC occurs in the groin region in a minority of patients. In one study made on patients diagnosed to have BCC, between the years 2007 and 2017, the occurrence in the groin was estimated at 0.094% [3]. Another larger study reported an incidence of 0.27% [4].

One of the first studies on this topic, studying 51 cases of BCCs occulting in the genitals and buttocks, was conducted by Gibson and Ahmed [4]. In this series, BCC in this location represented 0.27% of all cases of diagnosed BCCs. Histological and clinical presentations were highly heterogeneous and the average age was 73 years old at the moment of diagnosis [4].

Genetic factors play an important role in the development of BCC and can explain the existence of its many subtypes [5].

However, other etiological factors can explain the occurrence of this neoplasm in non-sun-exposed areas, such as in the groin. Among these, we could find local trauma and aging, artificial ionizing radiation, arsenic, chronic inflammation, hamartomas, and immune deficiency [6].

In our case, advanced age seems to be the principal recognized etiological factor.

Histologically, the most frequent subtypes described on the groin region were nodular (as in our reported case), superﬁcial and inﬁltrative [3,4].

A new clinicopathological variant of the BCC was described in 1999 by Megahed; the polypoid variant is characterized by a distinctive polypoid shape of the lesion, with the existence of a stalk attaching the proliferation to the skin surface [7]. This variant seems to occur commonly in women and in the scalp, buttocks, and genital regions [2,4,8,9].

When located in the groin, BCC presents no tendency to aggressive behavior, compared to BCCs occurring elsewhere in the body [3].

In a study made by Sexton et al., the frequency of the nodular and superficial subtypes has led to belief in the eventuality of slower growth of the BCC in non-sun-exposed areas [10].

Appropriate treatment can be guided by the pathological characteristics of BCCs and is essentially based on surgical resection with free margins [11,12].

## Conclusions

Basal cell carcinoma has been reported to occur in many rare locations. The groin region, as reported for our case, is one of these rare locations. More studies are needed to identify any prognostic implications of rare location for basal cell carcinomas. 

## References

[1] Hasan A, Kandil AM, Al-Ghamdi HS (2023). Sun-exposed versus sun-protected cutaneous basal cell carcinoma: clinico-pathological profile and p16 immunostaining. Diagnostics (Basel).

[2] Wu SE, YH Chen, HW Gao, CP Chiang (2019). Polypoid basal cell carcinoma on the right groin: a case report and review of literature. J Med Sci.

[3] Tidwell WJ, Sutton A, Gilbertson R, Humberson C, Greenway H (2019). Basal cell carcinoma of the groin and buttocks: a clinical and histological analysis of a rare presentation of a common tumor. Int J Dermatol.

[4] Gibson GE, Ahmed I (2001). Perianal and genital basal cell carcinoma: a clinicopathologic review of 51 cases. J Am Acad Dermatol.

[5] Raasch BA, Buettner PG, Garbe C (2006). Basal cell carcinoma: histological classification and body-site distribution. Br J Dermatol.

[6] de Giorgi V, Salvini C, Massi D, Raspollini MR, Carli P (2005). Vulvar basal cell carcinoma: retrospective study and review of literature. Gynecol Oncol.

[7] Megahed M (1999). Polypoid basal cell carcinoma: a new clinicopathological variant. Br J Dermatol.

[8] Mohan SV, Chang AL (2014). Advanced basal cell carcinoma: epidemiology and therapeutic innovations. Curr Dermatol Rep.

[9] Mansh M, Katz KA, Linos E, Chren MM, Arron S (2015). Association of skin cancer and indoor tanning in sexual minority men and women. JAMA Dermatol.

[10] Sexton M, Jones DB, Maloney ME (1999). Histologic pattern analysis of basal cell carcinoma: study of a series of 1039 consecutive neoplasms. J Am Acad Dermatol.

[11] Crowson AN (2006). Basal cell carcinoma: biology, morphology and clinical implications. Mod Pathol.

[12] Rubin AI, Chen EH, Ratner D (2005). Basal-cell carcinoma. N Engl J Med.

